# Frequency of early remodeling of left ventricle and its comparison between patients with stroke volume ≥97 Ml versus patients with stroke volume <97 Ml after aortic valve replacement for severe aortic regurgitation

**DOI:** 10.12669/pjms.326.11173

**Published:** 2016

**Authors:** Hafiz Muhammad Farhan Ali Rizvi, Zaigham Rasool Khalid, Allah Baksh, Mirza Ahmad Raza Baig

**Affiliations:** 1 Hafiz Muhammad Farhan Ali Rizvi, FCPS CS. Senior Registrar Cardiac Surgery, Pakistan; 2 Zaigham Rasool Khalid, FCPS CS. Senior Registrar Pediatric Cardiac Surgery, Pakistan; 3 Allah Baksh, FCPS CS. Senior Registrar Cardiac Surgery, Pakistan; 4 Mirza Ahmad Raza Baig, B.SC Hons., CPT. Clinical Perfusionist, Pakistan

**Keywords:** Aortic Regurgitation, Aortic Valve Replacement, Left Ventricular Remodeling

## Abstract

**Objectives:**

To evaluate the frequency of early remodeling in patients of severe aortic regurgitation after aortic valve replacement and to see the incidence of early remodeling in patients with stroke volume >97 ml versus < 97 ml before aortic valve replacement.

**Method:**

This was a prospective comparative study conducted from August 2013 to December 2014 in a tertiary care hospital. Fifty seven (57) patients of isolated chronic aortic regurgitation were included in this study. SPSS v23 was used for data analysis. Independent sample t-test was used for analysis of continuous variables and chi-square test for qualitative variables.

**Results:**

Out of fifty seven patients, early remodeling occurred in 34 (59.64%) patients after surgery. The mean pre-operative stroke volume of patient in whom remodeling occurred was 110.3+9.66 ml while mean pre-operative stroke volume of patients who did not undergo remodeling was 86.65+7.63 ml. There were 28 (82.4%) patients with stroke volume >97 ml in whom Remodeling occurred where as in patients with stroke volume <97 ml remodeling occurred only in 6 (17.6%) patients (p value 0.004). There was no in-hospital mortality.

**Conclusion:**

There is an association between stroke volume and early LV remodeling after Aortic valve replacement. Stroke volume >97 ml is a good predictor of early LV remodeling.

## INTRODUCTION

Aortic regurgitation (AR) is defined as reflux of blood from aorta into the left ventricle during diastole because of non-cooptation of aortic valve leaflets during diastole. Pure chronic aortic regurgitation is a rare disease. In the long run, it results in both pressure and volume overloaded conditions.^1^ In the initial stage, left ventricular (LV) dilatation and hypertrophy occurs to compensate volume overload and to keep the LV function normal.^2^ But progressive dilatation of LV is associated with greater threats of symptoms of LV dysfunction and sudden death after surgery.^3^-^5^ Aortic valve replacement surgery is advised in symptomatic patients of chronic AR. Many of these patients have improvement in symptoms after surgery; others develop post-operative heart failure and death. So there is a need to develop a reliable pre-operative predictor of post-operative LV systolic function so that appropriate timing of AVR could be established.

Left ventricular end diastolic dimension (LVEDD) and systolic function are important factors for the timing of AVR. But there is no cut-off value of LVEDD that can be used as an indicator of surgery.^6^,^7^ LV ejection fraction has also been widely studied to detect post-op LV dysfunction, but pre-op EF did not correlate with post-op EF.^8^-^10^ But one study has concluded that pre-op ejection fraction could be a predictor of successful recovery after AVR.^11^ However some authors have concluded that pre-op stroke volume >97 ml is a good predictor of post-operative LV remodeling and outcomes.^12^ In this study we evaluated the frequency of early remodeling in patients of severe aortic regurgitation after aortic valve replacement. We also compared the incidence of early remodeling in patients with pre-operative stroke volume >97 ml versus < 97 ml before aortic valve replacement.

## METHODS

This was a prospective comparative study conducted from August 2013 to December 2014 in a tertiary care hospital. Fifty seven patients of isolated chronic aortic regurgitation who were planned to undergo aortic valve replacement were included in this study. Patients with acute aortic regurgitation, having other comorbidities along with AR, e.g. coronary artery disease, mitral valve disease, aortic stenosis were excluded. Approval of study was taken from the ethical committee of the hospital. Informed consent was taken from every patient after thorough explanation of study protocols. Data regarding pre-operative LVEDD, LV ejection fraction and Stroke volume was taken from the pre-op echocardiographic report of the patient. In all patients stroke volume was calculated by measuring the outflow tract diameter in the parasternal long axis view at the base of the aortic valve leaflet by a consultant cardiologist. Post-operative measurement of LVEDD was made by a consultant cardiologist seven days after the surgery. LVEDD reduction was taken as significant if reduction was more than or equal to 10% of its baseline value. Patients were divided into two groups on the basis of remodeling.

SPSS v23 was used for data analysis. Independent sample t-test was used for analysis of continuous variables and chi-square test for qualitative variables.

## RESULTS

A total number of 57 patients with chronic severe aortic regurgitation who underwent aortic valve replacement were included in the study. All had their echocardiographic evaluation seven days before surgery and in early post-operative period i.e. on 7^th^ day after surgery. Measurements included the stroke volume and Left ventricular end-diastolic diameter. Early remodeling occurred in 34 (59.64%) patients after surgery. Mean age in early remodeling group was 41.41±14.2 years while 39.2±13.6 years in patients without remodeling. Left ventricular end-diastolic dimensions (LVEDD) of patients before surgery in patients with remodeling were 64.91±9.02 and in patients without remodeling were 69.92±8.60. The mean pre-operative stroke volume of patient in whom remodeling occurred was 110.3±9.66 ml versus 86.65±7.63 ml in patients without remodeling. There were 28 (82.4%) patients with stroke volume ≥97 ml in whom Remodeling occurred where as in patients with stroke volume <97 ml remodeling occurred only in 6 (17.6%) patients (p value 0.004). There was no in-hospital mortality.

## DISCUSSION

The proper timing of AV replacement in patients of chronic severe AR is still controversial. Recent ACC/AHA guidelines for AVR do not recommend surgery in patients of AR with EF <50%, LVEDD >70 mm, and LVESD >50 mm.^13^ Turk et al operated patients with these parameters and concluded that AV replacement in these patients is associated with significant survival rate.^14^ According to Euro heart survey only 32% patients with LVEF <50% to 30% undergo AVR and only 3% with LVEF<30%.^15^ This lower rate may be due to poor operative outcomes in these patients. In our study, there was only 1.75% patient with EF <30%, and 24.61% patients with EF <50-30%.

Volume overload and pressure overload occur simultaneously in patients of chronic severe AR so LVEF and LVESD are used to measure to pumping strength of LV and LVEDD are used for the measurement of severity of volume overload.^16^ After AVR, LVEDD are usually reduced within a few days after operation, resulting in reduction in preload and a transient decrease of LVEF.^14^,^17^ In our study, we found significant reduction in LVEDD in patients in whom remodeling occurred, and no reduction in LVEDD in patients without remodeling.

Stroke volume in patients of severe AR, depends on regurgitation volume and on the pumping capacity of LV. Some studies revealed relationship of SV with the extent and presence of viable myocardium in patients of LV systolic dysfunction.^12^,^18^ In our study we have shown that pre-operative stroke volume accurately predicts Left ventricular remodeling in patients with severe pure aortic regurgitation after aortic valve replacement. Sénéchal et al proposed that SV ≥97ml is a good predictor of early LV remodeling after AV replacement. In that study, all patients in whom remodeling occurred were of stroke volume >97 ml.^12^ In our study, early remodeling occurred in 82.4% patients with stroke volume >97 ml. Our results are comparable to their study.

The dB/dt is used as a standard reference for the estimation of LV contractility.^19^,^20^ It has a strong correlation with post-op EF.^10^ According to Gold et al there is a strong correlation between stroke volume and maximum dB/dt.^21^ Some other studies have revealed the correlation between SV and other variables of LV functionality. These studies have concluded that variations in SV with dobutamine infusion is associated with LV remodeling and has a potential to evaluate operative results,^22^.^18^,^22^,^23^ According to these studies there is a direct linear association between stroke volume and myocardial viability and contractility reserves. In our study, we also evaluated that there is a good relationship between stroke volume and early LV remodeling after AVR and stroke volume >97 ml is good predictor of early remodeling.

**Table-I:** Comparison of baseline and echocardiographic characteristics.

Name of Variable	(Patients with Early Remodeling)	(Patients Without Early Remodeling)	P-Value
Number of Patients	34	23	
Age (Y)	41.41±14.20	39.22±13.64	0.55
Male Gender (%)	24 (70.58)	13 (56.52)	0.19
BMI (kg/m^2^)	24.80±4.47	23.04±4.47	0.15
Diabetic History (%)	3 (8.6%)	2 (9.1%)	0.94
Hypertension History (%)	8 (22.9%)	3 (13.6%)	0.39
Pre-op Stroke Volume (ml)	110.38±9.66	86.65±7.63	<0.001
Pre-op LVEDD (mm)	64.91±9.02	69.92±8.60	0.04
Post-op LVEDD (mm)	57.72±8.46	68.57±9.08	<0.001
LV Ejection Fraction	56.32±8.90	55.81±10.62	0.85

**Table-II:** Stroke Volume and its relationship with remodeling.

Category of Stroke Volume	Patients with Remodeling	Patients without Remodeling	P-Value
S.V > 97 ml	28 (82. 4%)	9 (39.1%)	0.004
S.V <97 ml	6 (17.6%)	14 (60.9%)	

**Table-III:** Comparison of Pro-Op and Post-Op LVEDD.

	Pre-op LVEDD	Post-op LVEDD	P-value
Patients with Remodeling	64.91±9.02	57.72±8.46	<0.001
Patients without Remodeling	69.92±8.60	68.57±9.08	0.47

## CONCLUSION

There is an association between stroke volume and early LV remodeling after Aortic valve replacement. Stroke volume >97 ml is a good predictor of early LV remodeling.
